# A Randomized Controlled Trial of Resection Versus Thermal Ablation of Colorectal Cancer Liver Metastases (New Comet): Study Protocol

**DOI:** 10.1245/s10434-025-17984-5

**Published:** 2025-08-26

**Authors:** Ingrid Schrøder Hansen, Karina Roheim Lappen, Kristoffer Watten Brudvik, Bård Ingvald Røsok, Ida Björk, Daniel Østergaard, Marit Helen Andersen, Egidijus Pelanis, Andreas Westenvik Espinoza, Per Steinar Halvorsen, Sheraz Yaqub, Kristoffer Lassen, Bjørn von Gohren Edwin, Trygve Syversveen, Åsmund Avdem Fretland

**Affiliations:** 1 The Intervention Centre, Oslo University Hospital, Oslo, Norway; 2 Department of Hepato-Pancreato-Biliary Surgery, Oslo University Hospital, Oslo, Norway; 3 Institute of Clinical Medicine, University of Oslo, Oslo, Norway; 4 Department of Research and Development, Divisions of Emergencies and Critical Care, Oslo University Hospital, Oslo, Norway; 5 Department of Radiology, Oslo University Hospital, Oslo, Norway; 6 Department of Transplantation Medicine, Oslo University Hospital, Oslo, Norway; 7 Department of Interdisciplinary Health Sciences, University of Oslo, Oslo, Norway; 8 Department of Clinical Medicine, University of Tromsø, Tromsø, Norway

## Abstract

**Background:**

The use of thermal ablation of liver tumors is rapidly increasing. This is despite a lack of high-level evidence of the oncologic efficacy of ablation. Ablation is most often used in cases where resection is not possible, but as the technique has improved it is increasingly used as a substitute for resection.

**Patients and Methods:**

New Comet is an international multicenter randomized controlled double-blinded, non-inferiority trial comparing thermal ablation to resection of colorectal liver metastases. It will include 230 patients with colorectal liver metastases (up to 5 lesions, tumor size up to 3 cm) where thermal ablation and resection both are considered suitable treatment options for all lesions. The patients are randomized to either resection or ablation, and all lesions in each patient are treated with the same modality. Complete ablation will be verified with intraoperative computed tomography and ablation confirmation software.

**Conclusions:**

The primary endpoint is local tumor recurrence at 12 months. Secondary endpoints include overall survival, disease-free survival, time to surgical failure, health-related quality of life, complications, and cost-effectiveness (trial registration no.: NCT05129787).

**Supplementary Information:**

The online version contains supplementary material available at 10.1245/s10434-025-17984-5.

Colorectal cancer is on the rise in the Western world, with 160,000 new cases and a 2% annual increase in people under 50 years in the USA.^1^ Low- and middle-income countries also see an increase in colorectal cancer cases, increasing the global burden of this disease.^2^ The liver is the dominant site for colorectal cancer metastases. Long-term survival after surgery for colorectal liver metastases has improved dramatically, from less than 40% 5-year survival in the 1980s to more than 55% in recent reports.^3,4^ This improvement is related to advances in oncologic treatment, better preoperative diagnostics and patient selection, improved perioperative care and surgical equipment, and better anatomic and physiological understanding of the liver. Minimally invasive treatments now allow some patients to leave the hospital on the first day after surgery, without any detriment in oncologic outcomes.^5^

As more patients survive stage 4 colorectal cancer, the focus on reducing side effects of treatment has increased. Our group have previously shown that laparoscopic liver surgery improved patient’s postoperative quality of life compared with open liver surgery.^6^ In parallel, even less invasive treatments of liver tumors have emerged, such as thermal ablation.

A 2019 systematic review reaffirmed surgical resection as the gold standard for the treatment of colorectal liver metastases, concluding that resection should not be replaced by thermal ablation.^7^ In most published studies, ablation was associated with inferior oncologic outcomes compared with resection. However, the authors noted that these findings are often confounded by selection bias, as patients undergoing ablation were typically those with poorer prognostic factors; either deemed unresectable or too frail to undergo resection.

The use of ablation of liver tumors is rapidly increasing worldwide.^8,9^ This is despite a lack of high-level evidence of the oncologic efficacy of ablation of colorectal liver metastases. Ablation is most often used in cases where resection is not possible, but as the technique has improved it is increasingly used as a substitute for resection.^10^ The European multicenter prospective cohort trial MAVERRIC showed that microwave ablation performed in high-quality centers could lead to similar overall survival as surgical resection in small resectable colorectal liver metastases.^11^

The randomized controlled multicenter COLLISION trial was recently published.^12^ COLLISION was a noninferiority trial including 300 patients with 1–10 target colorectal liver metastases, defined as tumors that were considered both ablatable and resectable. The trial also included patients who had additional nontarget lesions that were only suited for one treatment modality, and approximately 27% of the patients in the trial were treated with a combination of resection and ablation. The primary endpoint of COLLISION was overall survival (OS), and OS following ablation was noninferior to that following resection. Local recurrence (frequently defined as new tumor tissue adjacent to the ablation zone or resection bed) was per patient 14% after resection and 12% after ablation. The local recurrence result was surprising to many, given that previous studies have reported local recurrence after surgery as low as 4%.^13^

Local recurrence after ablation is often related to incomplete ablation. Ablation confirmation software can improve the evaluation of ablation zones.^14^ The software uses co-registration of pre- and post-ablation computed tomography (CT) scans to illustrate how the ablation zone covers the tumor in all planes. Used intraoperatively it can increase precision when performing re-ablation by identifying inadequate margins. In the COLLISION trial ablation confirmation software was recommended, but not mandatory.

New Comet will be the first randomized trial to compare ablation alone with resection alone in patients with colorectal liver metastases. It will also be the first trial to use confirmation software in all ablation patients to optimize ablation margins where possible.

## Patients and Methods

### Study Design

New Comet is a multicenter randomized controlled trial. The study is powered to determine whether thermal ablation of colorectal liver metastases is non-inferior to resection with regard to local tumor recurrence at 12 months.

This is an ongoing trial at Oslo University Hospital (principal investigator, Oslo, Norway), Karolinska University Hospital (Stockholm, Sweden), University Hospital of North Norway (Tromsø, Norway), and Sahlgrenska University Hospital (Gothenburg, Sweden).

### Endpoints

#### Primary Endpoint

The primary endpoint of the trial is local tumor progression within 12 months. Local tumor recurrence is defined as new tumor tissue adjacent (0 mm distance) to the resection margin or ablation zone at the first imaging where the new tumor is visible.

#### Secondary Endpoints

- Overall survival (OS)
  - The main analysis will be on intention-to-treat (ITT) basis.
- A secondary OS analysis will be performed on an oncological homogenous group of patients with the following requirements:
  - no previous surgical procedure on the liver
  - no extrahepatic metastases
  - primary tumor resected
- Disease-free survival and liver-specific disease-free survival
- Time to surgical failure^15^
- Local tumor recurrence at 24 months
- Postoperative complications
- Health-related quality of life
- Cost-effectiveness
- Time to functional recovery
- Inflammatory response
- Intraoperative circulation physiology
- Evaluation of liver movement in different ventilation modes
- Minimum ablative margin needed to prevent local tumor recurrence
- Effect of blinding on patient-reported postoperative outcomes and hospital stay

**Fig. 1:**
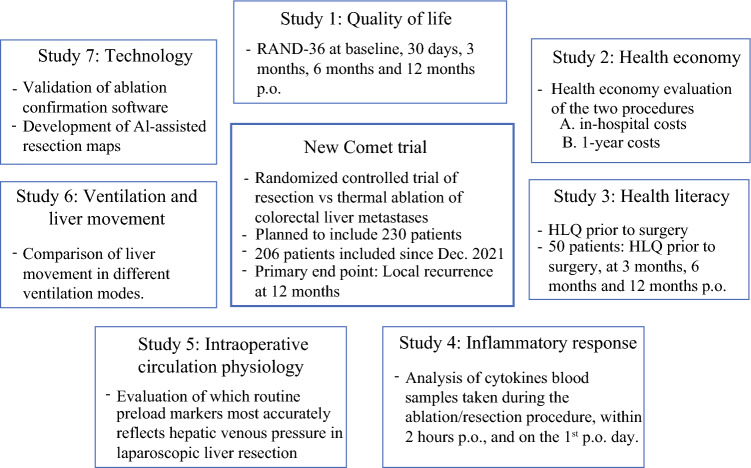
New Comet trial: overview of substudies; *RAND-36* RAND-36 Measure of Health-Related Quality of Life, *p.o.* postoperative; *HLQ* health literacy questionnaire, *AI* artificial intelligence

The New Comet trial has multiple substudies, illustrated in Fig. 1.

### Study Population

Patients referred for surgical treatment of colorectal cancer liver metastases to participating hospitals will be discussed in a multidisciplinary team with respect to surgical strategy. The trial will only include patients in whom all liver metastases can safely be treated with both modalities (either ablation only or resection only). Full inclusion and exclusion criteria are listed in Table 1.

**Table 1:** Inclusion and exclusion criteria

Inclusion criteria	Exclusion criteria
Histologically verified colorectal cancer. Colorectal liver metastases (proven or suspected) eligible for radical treatment using EITHER resection OR ablation (not a combination), as decided by the liver MDT meeting at the study center. Patient is fit to undergo both resection and ablation of all liver metastases. Size of largest lesion up to and including 30 mm. In case of solitary metastasis, resection plan includes resection of ≤ 2 anatomical segments. In case of multiple metastases in one continuous resection, resection plan can include ≤ 4 anatomical segments (including hemihepatectomy). ≤ 5 tumors to be treated in one procedure. Primary tumor either resected (primary first) or with a plan for curative treatment (liver first). Age ≥ 18 years.	More than 3 lung metastases. If solitary lung metastasis, size > 15 mm. If 2–3 lung metastases, size > 10 mm. (OR: unresectable lung metastases as decided by the lung MDT meeting). Presence of extrahepatic, extrapulmonary metastases. Surgical indication for removal of enlarged lymph nodes in the hepatic hilum. (Enlarged lymph nodes without indication of removal are not considered an exclusion criterium). Tumor closer than 10 mm to right/left main bile duct. Suspected tumor infiltration to adjacent organs. Progression (as of RECIST^25^) on second-line chemotherapy. Vanished lesions following neoadjuvant chemotherapy (Exception: Lesions with complete response to neoadjuvant chemotherapy; defined as no recurrence of lesion within 1 years after vanishing, including a chemo-free interval of at least 3 months). Previous inclusion in this trial. Not eligible for workup according to study criteria. Contraindication to contrast enhanced CT scan. Manifest liver cirrhosis with Child–Pugh score ≥ B7. Pregnancy. ECOG performance status ≥ 3. Simultaneous resection of primary tumor or any other concomitant surgical procedure, with the exception of cholecystectomy. Need for liver volume expansion. Any other reason why, in the investigator’s opinion, the patient should not be included.

MDT, multidisciplinary team; RECIST, response evaluation criteria in solid tumors; CT, computed tomography; ECOG, Eastern Cooperative Oncology Group

### Preoperative Workup

All patients will undergo standardized radiological workup before inclusion, consisting of contrast-enhanced X-ray computed tomography (CT) scans of the chest and abdomen, and magnetic resonance imaging (MRI) of the liver enhanced with liver-specific contrast agent. The last CT or MRI of the liver taken before randomization must not be more than 5 weeks old at the date of surgery.

### Randomization

Randomization will be electronically performed after all neoadjuvant treatment is administered if applicable, inclusion and exclusion criteria are controlled, and informed patient consent has been obtained. The patients will be allocated to treatment with resection or ablation with an allocation ratio of 1:1. A dedicated electronic randomization software has been created using block randomization with variable block sizes and stratified randomization to ensure similar numbers of the following prognostic factors in both groups:

- Size of largest tumor over 2 cm.
- Superficial lesion (no visible liver tissue between tumor and liver surface on preoperative radiology).
- Treatment center.

### Sample Size Calculation

Ablation has previously been associated with high local recurrence rate, up to 20%, compared with 2–3% for resection.^13^ Recent analyses of ablation using modern techniques for needle guidance and ablation zone confirmation report local recurrence as low as 4%.^14^

In this trial we expect a local recurrence rate of 2% in the resection group (control) and 7% in the ablation group (intervention). Given that ablation has frequently been used despite expected local recurrence numbers of up to 20%, we consider a non-inferiority margin of 13% acceptable. With a two-sided alpha of 5% and a power of 80%, 208 patients are needed to exclude a difference in favor of the resection group of more than 13 percentage points (equaling a local recurrence percentage of 2% following resection and 15% following ablation). To allow for 10% dropout we aim to include 230 patients in total, 115 in each group.

### Study Procedures

#### Resection (Standard)

Resection can be performed as laparoscopic surgery, robot-assisted surgery, open surgery, or a combination of these techniques. An adequate resection margin is considered to be more than 1 mm of healthy liver tissue on the resected tumor, except in cases where the tumor is divested from a vessel, so called “R1 vascular”. The resections will be performed using the technique preferred by the responsible surgeon. Parenchyma transection will most frequently be performed using ultrasound guidance and a bipolar sealer transection technique assisted by ultrasonic aspirators and endo-staplers if needed.

#### Ablation (Intervention)

Ablation can be performed percutaneously, laparoscopically, with robotic assistance, through an open approach, or a combination of these techniques. Needle placement is guided by either computed tomography or ultrasound. Microwave ablation is preferred, although radiofrequency ablation is acceptable. All procedures are conducted under general anesthesia.

### Ablation Confirmation

Perioperative CT scanning will be performed in all patients randomly assigned to ablation. A CT scan will be performed with the patient in general anesthesia and with the patient positioned for the ablation procedure. Another CT scan will be performed after completion of ablation, and ablation margins will be evaluated intraoperatively using ablation confirmation software. We aim to achieve an ablation margin of 10 mm, but a margin of minimum 5 mm will be accepted. The exceptions to this are cases where the ablated tumor is close to a vessel, where the adequate margin must be a matter of individual judgement and sometimes may be less than 5 mm.

CT scanning for confirmation of ablation margin can be performed later during the same in-patient visit if renal function or other medical reason prohibits additional intraoperative contrast enhanced CT scanning, or if laparoscopic ablation is performed in an operating room without CT. If insufficient ablation margin is detected, defined as above, re-ablation should be performed within 1 week.

### Postoperative Care

Postoperative care will be similar in both arms and based on enhanced recovery principles, which include early mobilization and expanding oral intake as desired by the patient. Patients will be evaluated for adjuvant chemotherapy at their local oncologic department, according to national guidelines.

#### Treatment of Cancer Recurrence

Treatment of cancer recurrence or progression should follow national guidelines and be decided by a multidisciplinary team. However, local tumor recurrence after ablation will preferably be treated with new ablation, provided approval from the local multidisciplinary team.

In case of new liver metastases, the multidisciplinary team will evaluate the best treatment option for the patient. In cases where resection and ablation are considered equivalent, the treatment following initial randomization should be preferred.

#### Follow-Up

The patient will be contacted by the study center 30 days postoperatively for registration of complications, quality of life, health economy, referral to oncologist, and unblinding.

**Fig. 2:**
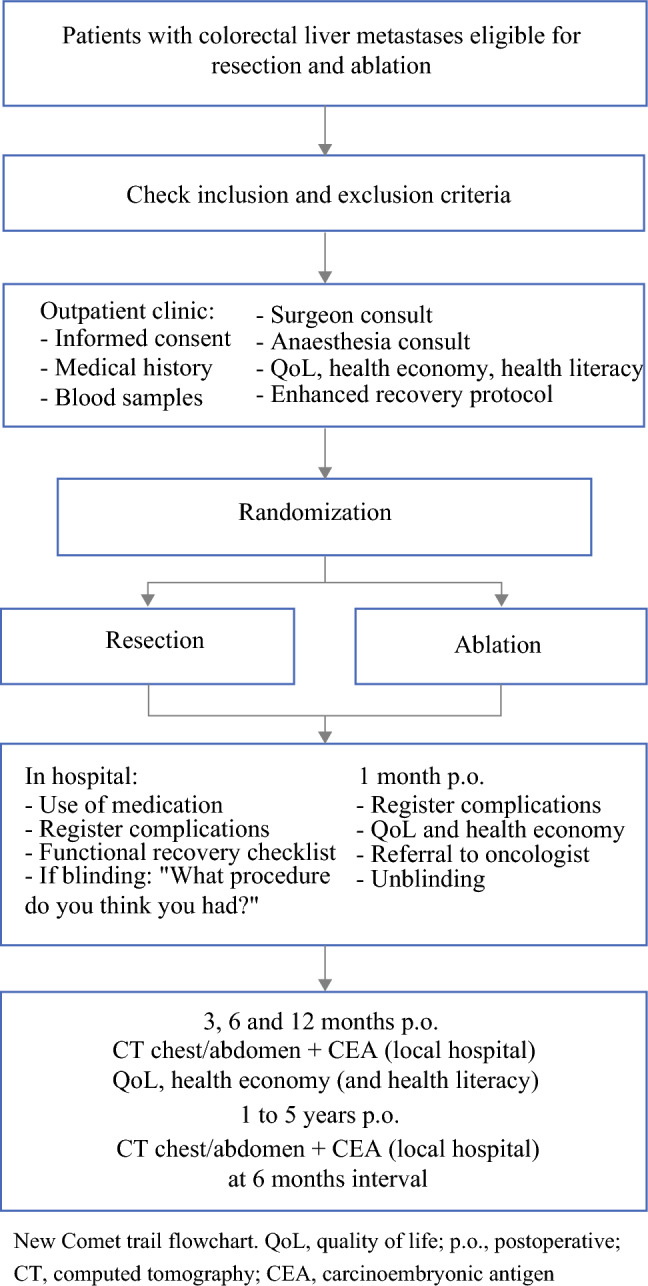
New Comet trial flowchart^26^

Routine follow-up will be at 3 months, 6 months, and 12 months postoperatively (Fig. 2). At each of these CT scans of the chest, abdomen, and pelvis; clinical control; and measurement of carcinoembryonic antigen (CEA) will be performed. Quality of life and health economy will be evaluated.

After completion of follow-up for the primary endpoint (local tumor recurrence), the patients will undergo standard follow-up according to national guidelines. This consists of CT scans of the chest, abdomen, and pelvis; clinical control; and measurement of CEA every 6 months. Additional follow-ups are allowed if clinically indicated, or according to local guidelines provided that planned follow-up is not systematically different between groups.

Any cancer recurrence will be evaluated and verified by an experienced radiologist at the treatment center. A centralized radiology review board will evaluate any suspected local tumor recurrence. Radiologists cannot evaluate a local recurrence following ablations they have performed themselves. In case of unresolved decisions, imaging will be transferred to an external expert center for a final decision.

#### Double Blinding

**Fig. 3:**
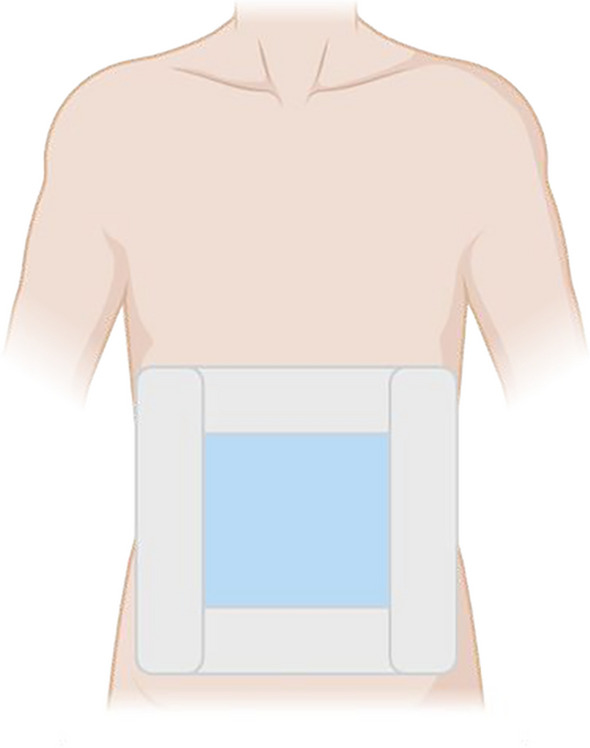
Large abdominal dressing ^27^

Patients and medical and nursing staff will be blinded for the type of intervention. At the end of surgery patients will receive a securely taped, 40 cm × 40 cm abdominal dressing to mask their incisions or percutaneous puncture wounds (Fig. 3). This abdominal dressing can be removed when all criteria for functional recovery are met, or at the latest postoperative day 3. Staff will be encouraged not to read digital operation reports until unblinding; but in case of medical need, unblinding by reading digital operation reports is allowed. The surgeon in charge of the patient in the postoperative setting is allowed to unblind themselves for any medical reason. Similarly, all involved healthcare personnel will be unblinded as the patient leaves the hospital (to prepare appropriate discharge reports). The patient will be informed of what treatment they received 30 days postoperatively.

At Oslo University Hospital double blinding will be performed, while it will not be mandatory in the multicenter setting. It will be up to each center to determine whether blinding is feasible at their institution. However, blinding is encouraged.

#### Data Collection and Monitoring

Baseline characteristics will be recorded before randomization. The required clinical data, i.e., primary and secondary outcomes, will be collected after randomization. All complications will be scored using the Clavien–Dindo score of surgical complications^16^ and the Comprehensive Complication Index.^17^

Follow-up data will be collected from the local hospital or general practitioner by obtaining copies of patient medical records and CT scans. For long-time follow-up data, records will be collected for up to 10 years.

#### Health-Related Quality of Life

Patients will be asked to complete the RAND-36 questionnaire before study intervention and after 1, 3, 6, and 12 months.

#### Cost-Effectiveness

Direct and indirect healthcare costs will be collected in a 12-month perspective after the intervention.

Further details on data collection for health literacy and anesthesiologic substudies can be found in supplementary appendix.

#### Statistical Analysis

The primary outcome of local tumor recurrence within 12 months after study intervention will be analyzed with a Kaplan–Meier plot, a log-rank test for equality of survival curves, and a Cox proportional hazard regression model. The primary analysis will be the Cox regression model, and we will report the estimated hazard ratio (HR) with a 95% confidence interval for thermal ablation versus surgical resection and a p-value for the null hypothesis of no treatment difference (HR 1). The median survival time in each treatment group will be estimated with the Kaplan–Meier estimator and presented with 95% confidence intervals. The outcomes of disease-free and overall survival will be analysed and reported in the same manner as the primary outcome.

Continuous outcomes measured at one time point (i.e. at 12 months) will be analysed with two-sample *t*-test, with adjustments for unequal variances (the Welch *U* test), and 95% confidence interval for the difference between means. In cases where the distribution of the outcome is considerably skewed, median regression will be used and differences in medians instead of means will be analyzed.

Change in continuous outcomes measured at baseline and 12 months will be analyzed with analysis of covariance (ANCOVA), i.e., a linear regression model where the 12 months measurement is the dependent variable and treatment and baseline measurement are the independent variables. The between-treatment difference in change from baseline will be estimated with a 95% confidence interval, and a *p*-value for the null hypothesis of no treatment difference.

Dichotomous variables will be analyzed with Fisher mid-*p* tests and Newcombe hybrid score confidence intervals.^18^ Ordered categorical variables with more than two categories will be analyzed with Wilcoxon–Mann–Whitney tests (score tests for effect in a proportional odds model).^19^ A statistical analysis plan will be prepared and published at clinicaltrials.gov.

#### Funding and Ethics Approval

The study is funded by the South-East Norwegian healthcare authority (Helse Sør-Øst) through an open project support grant (2021-065). The funding body has no impact on the scientific design of the study. Approval was obtained from the Regional Committee for Health and Research Ethics (2021/255384/REK Sør-Øst A) and from the Data Protection Official for Research at Oslo University Hospital. The study was registered at clinicaltrials.gov (NCT05129787) before recruitment started. Ethical approval has been obtained for all study sites.

The treatment of the patients in this study will be as part of routine treatment offered to patients with colorectal cancer liver metastases. Informed consent will be obtained from patients that participate in the trial, following good clinical practice guidelines.

#### Safety

No interim-analysis will be performed. Serious adverse events (SAEs) must be reported by the investigator to the sponsor within 24 hours after the site has gained knowledge of the event. The serious adverse event will be reviewed by the sponsor designee, who will consider the causality and expectedness of the event. Relapse and death due to cancer progression and hospitalizations for elective treatment of a preexisting condition do not need reporting as a serious adverse event. If the sponsor designated clinician reports a worrisome trend in SAE, a data management committee (DMC) will be appointed. The DMC will review SAE outcomes by group and could advise the study to be stopped.

## Discussion

This trial is designed to assess whether local tumor recurrence following thermal ablation with ablation confirmation software is non-inferior to surgical resection in colorectal liver metastases measuring up to 3 cm.

Local tumor recurrence was chosen as primary endpoint because this has historically been considered the major limitation of thermal ablation. We expect the incidence of local tumor recurrence to decline with introduction of ablation confirmation software. The recently published COVER-ALL study showed that use of software-based assessment during thermal ablation significantly improved the minimal ablative margin compared with visual assessment, and reduced the rate of local tumor recurrence from 15 to 4%.^14^

The New Comet trial has a relatively high non-inferiority margin of 13%. We consider this acceptable as re-ablation of local recurrence is likely to be a minor procedure to these patients. A difference in local tumor recurrence rate is not likely to affect overall survival, and the MAVERRIC trial showed similar survival after resection and ablation despite a rate of 23% local tumor recurrence per patient within 1 year after ablation.^11^ The non-inferiority margin was thoroughly discussed within the study group and with an international group of experts.

Overall survival was not chosen as primary endpoint, as randomized trials are challenging when the expected difference between groups is small. The MAVERRIC trial indicated that microwave ablation has similar overall survival as resection, and these results were validated in the randomized COLLISION trial, although with hazard ratio of 1.30 as upper limit for non-inferiority.

The New Comet trial has several limitations. The trial includes patients who have been previously treated with liver resections and/or ablation. Furthermore, a potential recurrence might be treated with a different modality than the trial randomization. The study centers are Scandinavian with similar healthcare systems. All centers have surgeons experienced in minimal invasive liver surgery and interventional radiologists experienced in percutaneous ablations. The trial will compare ablation and resection in expert hands with advanced technology, and thus limit generalizability to other centers.

Most patients undergoing surgery for colorectal liver metastases will develop recurrent disease.^13,20^ Although surgery is intended to be curative, its benefit is limited in cases of early, inoperable recurrence. This underscores the value of minimally invasive approaches, which may reduce morbidity and facilitate repeat interventions when needed. Minimally invasive parenchyma-sparing resection is a well-established treatment for colorectal liver metastases,^21^ aiming to achieve local tumor control with minimal surgical trauma. Overall survival and risk of recurrence are more closely correlated to the biology of the disease than resection margins, and more extensive resections do not reduce the recurrence rate or improve survival.^20,22–24^ Ablation can be a continuation of this minimally invasive parenchyma sparing concept, provided it achieves local tumor control comparable to that of resection.

Results after thermal ablation have previously been highly operator dependent. Software for ablation confirmation can potentially lead to reliable achievement of complete ablation, not only in expert hands or depending on which ablation system is used.

The New Comet trial compares ablation only to resection only. The aim is to test a strategy of ablating all tumors as first-line treatment to achieve local tumor control and allow time to assess the development and extent of the disease, an “ablate and wait”-strategy. This observation period may improve selection for major hepatectomy, which is still associated with substantial morbidity and mortality, and help avoid futile surgery. Liver transplantation has emerged as a treatment option for selected patients with colorectal liver metastases. Liver transplantation is highly extensive treatment that also has implication on organ allocation, and a better test of tumor biology is therefore needed. The New Comet trial might shed light on whether an “ablate and wait” approach could be part of a future strategy for patients with colorectal liver metastases.

## Supplementary Information

Below is the link to the electronic supplementary material.

Supplementary file1 (DOCX 22 kb)

Supplementary file2 (MP4 155252 kb)
