# Supplementary material for: A Randomized Controlled Trial of Resection Versus Thermal Ablation of Colorectal Cancer Liver Metastases (New Comet): Study Protocol
Source: Ann Surg Oncol. 2025 Aug 26;32(12):9146–53. doi: 10.1245/s10434-025-17984-5 (PMC12534323; doi:10.1245/s10434-025-17984-5)
Supplement: Supplementary file 1 — Supplementary file1 (DOCX 22 kb) [file 10434_2025_17984_MOESM1_ESM.docx]

**Supplementary appendix**

Supplement to: A randomized controlled trial of resection versus thermal ablation of colorectal cancer liver metastases (New Comet): Study protocol

## Substudies in the New Comet trial

### Health literacy

Health literacy (HL) can be defined as people’s ability “to access, understand, appraise, remember and use information about health and health care, for the health and well-being of themselves and those around them”^1^. The extent to which a patient understands and considers medical options, and their potential consequences, depends on their level of health literacy. Health literacy will be assessed in a cohort of the trial patients using the Health Literacy Questionnaire. Patients will be asked to complete this questionnaire prior to surgical treatment. A subset of 50 patients will be asked to complete additional questionnaires at 3, 6, and 12 months after study intervention, to assess changes in health literacy over time.

### Inflammatory response

Inflammation has been associated with elevated risk of cancer progression and metastatic capacity in colorectal cancer (CRC)^2,3^. The degree of inflammatory response will be evaluated by analysing a panel of cytokines and complement factors from blood samples collected before and during the procedure, 2 hours postoperatively and on the 1^st^ postoperative day.

### Haemolysis

Haemolysis may be observed after thermal ablation. The occurrence of haemolysis after thermal ablation will be investigated by analysis of classic haemolysis markers (Lactate Dehydrogenase, Bilirubin, Haptoglobin and free-Hemoglobin) preoperatively, within 2 hours postoperatively and on the first postoperative day.

### Intraoperative circulation physiology

A subgroup of 20 patients randomized to undergo resection will be asked to provide a separate written consent for a study of intraoperative circulation physiology. After induction of anaesthesia, invasive monitoring equipment will be placed to measure pressure in the hepatic vein and the transhepatic pressure. These values will be compared with different preload markers, including central venous pressure, pulmonary artery occlusion pressure, left ventricular end-diastolic volume, stroke volume variation and pulse pressure variation. Additionally cardiac output will be assessed using pulmonary artery catheter, pulse contour analysis (Flowtrach) and transthoracic echocardiography.

Measurements will be conducted before and after interventions involving:

- Patient position: Flat versus tilted head-up at 10 degrees
- Positive end-expiratory pressure (PEEP): 0 cm H_2_O versus 10 cm H_2_O
- Pneumoperitoneum: none versus 12 mmHg

### Evaluation of liver movement in different ventilation modes

Liver movement will be assessed in a subgroup of 20 patients randomized to undergo ablation. After induction of anaesthesia, the radiologist will measure and record a video of the movement of the left and right liver margin at different ventilation modes (jet ventilation with single and dual mode, one lung ventilation, gentle ventilation, normal ventilation). These videos will later be reviewed by a blinded radiologist for measurement of liver movement and assessment of interobserver variation.

1. World Health Organization *Health literacy development for the prevention and control of noncommunicable diseases: volume 1: overview*. Vol. 1. Publications. 2022. <https://creativecommons.org/licenses/by-nc-sa/3.0/igo>

2. Kraus S, Arber N. Inflammation and colorectal cancer. *Curr Opin Pharmacol*. Aug 2009;9(4):405-10. doi:10.1016/j.coph.2009.06.006

3. Lasry A, Zinger A, Ben-Neriah Y. Inflammatory networks underlying colorectal cancer. *Nat Immunol*. Mar 2016;17(3):230-40. doi:10.1038/ni.3384
